# Predicting Postcesarean Pain: A Prospective Cohort Study Using a 3-Question Questionnaire, Local Anesthesia Infiltration, and Observer Rating

**DOI:** 10.1155/prm/6903333

**Published:** 2025-04-21

**Authors:** Unyime S. Ituk, Sapna Ravindranath

**Affiliations:** ^1^Department of Anesthesia, University of Iowa, Iowa City, Iowa, USA; ^2^Department of Anesthesia, Indiana University, Indianapolis, Indiana, USA

## Abstract

**Purpose:** Acute postoperative pain is a typical complaint following cesarean delivery (CD). The current standard for postcesarean pain management is the use of a multimodal analgesia regimen which is beneficial for many but may be inadequate for some patients. This study aimed to determine if combining patients' response to a pain rating questionnaire, their pain score during local anesthetic infiltration (LAI) preceding spinal anesthesia for CD, and an anesthesiologist's prediction of postcesarean pain severity can predict the intensity of postcesarean pain.

**Methods:** This was a prospective study of ninety women undergoing scheduled CD under spinal anesthesia. Patients completed a pain rating questionnaire preoperatively and rated pain on LAI before spinal injection, and an anesthesiologist predicted the severity of postcesarean pain. Postoperative pain scores were assessed at rest and with movement at 6, 24, and 48 h after surgery.

**Results:** The patient's expected postoperative pain (*β* = 0.39, *p*=0.0011), perceived analgesic requirements (*β* = 0.34, *p*=0.0002), pain on LAI (*β* = 0.22, *p*=0.004), and anesthesiologist's predicted postoperative pain severity (*β* = 0.22, *p*=0.01) were associated with mean postoperative pain after CD. The multivariate model analysis found that the pain rating questionnaire and the an anesthesiologist's prediction of postcesarean pain severity contributed to postoperative pain modeling (*R*^2^ = 0.27).

**Conclusion:** Combining a preoperative pain rating questionnaire with an anesthesiologist's prediction of postcesarean pain severity accounted for 27% of the variance in mean postoperative pain with movement and may be a useful tool in predicting postcesarean pain.

**Implications:** This study highlights the potential of a combined preoperative pain rating questionnaire and anesthesiologist's predictions to improve postcesarean pain management. By accounting for 27% of the variance in mean postcesarean pain with movement, this approach could enhance pain management outcomes for CD patients.

## 1. Introduction

Cesarean delivery (CD) is the most common major surgical procedure in the United States, with approximately 1.3 million cases annually, accounting for 32% of all live births [1]. Acute postoperative pain is a common problem following CD, and along with intraoperative pain was ranked highest among undesirable outcomes for women undergoing CD [2]. The current standard for postcesarean pain management is the use of a multimodal analgesia regimen that consists of neuraxial opioids, oral nonsteroidal anti-inflammatory drugs, acetaminophen, and nurse-administered oral opioids for breakthrough pain [3]. Though beneficial for most women, this one-size-fits-all approach is likely inadequate for some. Inadequate analgesia can limit early ambulation, self-care, and the ability to care for the newborn and is significantly associated with the development of postpartum depression [4, 5], and the severity of acute postoperative pain is a significant predictor of future persistent or chronic surgical pain [4, 6]. Multiple factors contribute to post-CD pain and significant interindividual variability in pain perception [7]. Preoperatively identifying women at high risk for severe postoperative pain may facilitate a targeted analgesic approach to this population. Predicting postoperative pain intensity has been of interest in the past with a variety of preoperative measures being investigated for predicting the severity of pain and the development of chronic postsurgical pain. Previous studies have shown that a simple rating of the patient's anticipated pain and analgesic threshold correlates with postoperative pain scores and opioid consumption compared to individual-validated psychological questionnaires [8, 9]. Other studies have investigated the role of preoperative quantitative sensory tests (QSTs) in identifying patients at risk of severe acute postoperative pain [10–12]. However, QST requires specialized equipment, and the correlation with pain outcomes is inconsistent. In an observational trial by Orbach-Zinger et al., they reported that severe pain response during local anesthesia infiltration (LAI) before neuraxial anesthesia for CD was predictive of severe acute postoperative pain with a 92% sensitivity and 93% specificity and was associated with higher analgesic consumption [13]. Pain response to LAI is not technically a QST modality, but it is a routine step in initiating neuraxial anesthesia for CD and does not require additional equipment. However, it is unclear if the findings in the study are generalizable, especially in patients on a standardized scheduled analgesic regimen. Furthermore, the study by Orbach-Zinger et al. limited the assessment of outcome measures to the first 24 h after CD which does not capture most of the in-hospital postoperative period in this patient population.

Our primary aim was to determine if the combination of a patient's response to the simple pain rating questionnaire (expected postoperative pain, their anticipated analgesic threshold, and perceived analgesia requirements), their pain rating during LAI before spinal anesthesia for CD, and an anesthesiologist's prediction of postoperative pain can predict severe postcesarean pain. We hypothesized that combining these three tools would predict the severity of acute postoperative pain after CD.

## 2. Methods

This prospective observational study was conducted at the University of Iowa Hospitals and Clinics and approved by the University of Iowa Institutional Review Board in August 2018 (Human Subjects Office IRB #201808806). This study adheres to the Strengthening the Reporting of Observational Studies in Epidemiology (STROBE) guidelines [14]. The STROBE checklist is available in the Supporting Information. Healthy pregnant women aged ≥ 18 years, with a singleton term (≥ 37 weeks) gestation, nonlaboring, and scheduled for CD under spinal anesthesia were approached while in the preoperative area to participate in the study.

Exclusion criteria included nonfluency in English, ASA physical status ≥ III, onset of labor, emergency CD, contraindication to spinal anesthesia, failed spinal anesthesia requiring conversion to general anesthesia, if the patient received intravenous (IV) supplementation for analgesia or anesthesia during the CD, intolerance or allergy to opioids, nonsteroidal anti-inflammatory drugs or local anesthetics, and participation in another clinical study.

After obtaining written informed consent, maternal demographic data, including age, weight, height, and parity, were documented. Before proceeding to the operating room, recruited patients completed a three-question pain rating questionnaire. Specifically, the following questions were asked: (1) expected postoperative pain: “How much pain do you think you will experience after surgery, using a scale of 0–10?” (0 being “no pain” and 10 being “worst pain imaginable”); (2) anticipated threshold for analgesia: “At what point on a pain scale of 0–10 are you likely to request for medication to relieve your pain?” (0 being “no pain” and 10 being “worst pain imaginable”); and (3) perceived analgesic requirements: “How much medication for pain relief after surgery do you think you will need, on a scale of 0–10?” (0 being “no need for pain medication” and 10 being “highest need for pain medication”). Baseline preoperative pain scores at rest and with movement were assessed using a numerical verbal pain scale (NVPS), with 0 being “no pain” and 10 being “the worst possible pain.”

All patients received spinal anesthesia in the sitting position at L3-L4 or L4-L5 vertebral interspace. After routine skin preparation with chlorhexidine and draping, the anesthesiology resident infiltrated the skin overlying the chosen vertebral interspace with a local anesthetic. We used a standardized written script to describe to the patient that infiltration of the skin was about to occur: “I am going to inject the numbing medicine where I will place the spinal anesthetic.” The protocol for LAI was as follows: 3 mL of 1% lidocaine (not containing additives) was drawn up in a 3 mL syringe and injected using a 25 G × 1.5-inch needle. A total of 3 mL was injected with an initial intradermal wheal and then into deeper tissues. On completion of the LAI, a research support staff approached the patient after 20 s to rate their pain using a NVPS of 0–10 (with 0 being “no pain” and 10 being “worst possible pain”), that is, the pain experienced from the LAI. The anesthesia team was not aware of the value chosen by the patient on the NVPS response. The research support staff then approached the attending physician anesthesiologist supervising the resident to predict the anticipated severity of the patient's postoperative pain on a scale of 0–10 (with 0 being “no pain” and 10 being “worst possible pain”) based on their observation of the patient's reaction to LAI. Intrathecal 0.75% hyperbaric bupivacaine 12 mg, fentanyl 15 mcg, and preservative-free morphine 150 mcg were administered via a 25 G Whitacre spinal needle. A variable rate phenylephrine infusion was titrated to maintain systolic blood pressure within 20% of the baseline preoperative value. Eight attending physician anesthesiologists who provide care in the labor and delivery unit participated in predicting the postoperative pain severity and have between 5 and 30 years of obstetric anesthesia experience.

All women had a Pfannenstiel skin incision and a low uterine segment incision, and the uterus was exteriorized to repair the hysterotomy. On completion of surgery, IV ketorolac 30 mg was administered, and patients were transferred to the postanesthesia care unit (PACU). The management of postoperative analgesia was like what has been described in our previous study [15]. In the PACU, the patients received IV morphine 2 mg every 10 min up to a maximum dose of 10 mg as needed for numerical verbal pain score > 4.

The postoperative analgesia regimen was standardized: scheduled IV ketorolac 30 mg and oral acetaminophen 650 mg were administered every 6 h for the first 24 h. Oral ibuprofen 600 mg every 6 h was substituted for IV ketorolac after 24 h. Breakthrough pain was managed with oral oxycodone 5–10 mg at the patient's request. Patients with a NVPS of 4–6 received oxycodone 5 mg, and those with NVPS ≥ 7 received 10 mg. Patients received IV ondansetron 4 mg as a first-line antiemetic and IV promethazine 25 mg if there was a poor response to the first agent.

A research team member blinded to the responses on the three-question pain rating questionnaire and pain rating following LAI interviewed the patients using a standardized script at 6, 24, and 48 h postoperatively. Both baseline preoperative and postoperative pain scores at rest and with movement were assessed using a NVPS (with 0 being “no pain” and 10 being “the worst possible pain”). Pain at rest was assessed with the patient lying supine, and pain with movement was assessed when moving from the supine to an upright sitting position.

Patients were asked to rate the severity of postoperative nausea using an 11-point numerical rating scale of 0–10 (0 being “no nausea” and 10 being “worst nausea possible”). The number of vomiting episodes, if any, was documented during the 48-h study period. Pruritus was also assessed using an 11-point numerical rating scale (0 being “no pruritus” and 10 being “worst pruritus possible”). The time to first opioid request and total opioid consumption for each patient was also recorded. The primary outcome response measures were mean postoperative pain with movement in the first 48 h after CD. Other outcome measures of interest were time to first opioid request and opioid consumption. The time to first opioid request was defined as hours from the end of surgery. For analysis, all opioid intake was converted to IV morphine milligram equivalents (MMEs) using a conversion factor of 0.5 [16].

### 2.1. Statistical Analysis

Pan et al.'s study using the three simple preoperative screening questions reported that it accounts for 20% of the variance in postcesarean evoked pain [9]. We estimated that the three screening tools will account for 15% of the variance in postcesarean pain and a sample size of 80 patients will achieve 90% power with the Type I error rate of 5%. Taking into consideration potential protocol violations and drop-outs, the aim was to recruit 90 patients. Summary statistics were calculated for all patient's demographics and outcome measures of interest. Continuous variables are reported as means and standard deviations and counts and percentages. Assessments of pain, opioid use, and time to first opioid request were made using the generalized linear modeling (GLM) framework. Histograms of outcome measures were reviewed to ensure we specify an appropriate distribution when fitting the GLMs. For outcomes with skewed distribution, the slope estimates were obtained through modeling the log-transformed data and then exponentiated to represent the mean ratio. Point estimates, 95% confidence intervals, and *p* values were calculated for each univariate model predictor. Next, we considered all possible pairings of the significant univariate predictors (expected postoperative pain, perceived analgesic requirements, anesthesiologist's prediction of postoperative pain severity, pain score on LAI, and parity) for bivariate GLMs, without and with interaction effects. We compared the Akaike information criteria (AIC) (smaller indicates a better fit) across all models to identify the optimal bivariate predictor set for each outcome. We reported predictor estimates for only the best model according to AIC. All tests for predictor significance are evaluated at the *α* = 0.05 level. All data were analyzed using SAS Version 9.4 (SAS Institute Inc., Cary, North Carolina).

## 3. Results

The study was conducted between October 2018 and November 2020. One hundred and twenty-two women who met the eligibility criteria were approached to participate. Thirty-two patients declined, and 90 were enrolled and completed the study. The demographic and obstetric data of the study cohort are reported in Table 1.

The mean (SD) scores for expected postoperative pain, anticipated threshold for analgesia, and perceived analgesic requirements on the three-question pain rating questionnaire were 6.73 (1.69), 6.08 (1.77), and 5.77 (2.22), respectively. The mean (SD) NVPS on LAI was 3.81 (2.63). The mean (SD) predicted postoperative pain severity by attending anesthesiologists was 3.66 (2.21). Summarized in Table 2 are descriptive statistics of the predictor and outcome variables.

In the univariate model, the expected postoperative pain (Q1) (*β* = 0.39, 95% CI: [0.15, 0.62], *p*=0.0011), perceived analgesic requirements (Q3) (*β* = 0.34, 95% CI: [0.16, 0.51], *p*=0.0002), pain on LAI (*β* = 0.22, 95% CI: [0.07, 0.40], *p*=0.004), and anesthesiologist's prediction of postoperative pain severity (*β* = 0.22, 95% CI: [0.04, 0.41], *p*=0.01) were associated with mean postoperative pain with movement. The univariate model for predictors of mean postoperative pain with movement is reported in Table 3. The top multivariate model included the simple pain rating questionnaire (*β* = 1.204, *p* < 0.0001), the anesthesiologist's prediction of postoperative pain severity (*β* = 1.138, *p*=0.006), and their interaction (*β* = −0.157, *p*=0.02). Both were significant predictors of mean postoperative pain with movement in the first 48 h after CD (*R*^2^ = 0.27). The slope estimates represent the expected difference in mean pain as we increase our predictor by one unit. The top multivariate model for predictors of pain with movement after CD is summarized in Table 4. None of the predictors were associated with the time to first opioid request or opioid consumption at any time point during the study.

## 4. Discussion

The findings of our study show that the combination of a pain rating questionnaire and the anesthesiologist's prediction of postcesarean pain severity accounted for 27% of the variance in mean postoperative pain with movement. Our study's effect size is smaller than that reported by Carvalho et al. in a previous study [8]. They reported that the three simple ratings of patients' expected postoperative pain, anticipated analgesic threshold, and perceived analgesic needs determined before CD predicted 45% of the variability in postoperative pain and 21% of opioid consumption. The authors combined pain at rest and pain with movement into a single outcome measure which may have contributed to the difference in effect size. We chose pain with movement as the primary outcome because it is more commonly experienced during routine daily activities and likely has a greater adverse impact on recovery compared to pain at rest. Furthermore, the patients in our study received a higher dose of intrathecal morphine during spinal anesthesia, which is associated with a longer duration of analgesia [17]. In another study by Pan et al., they reported that the three simple perioperative screening questions on surgical anxiety, anticipated pain, and anticipated analgesic requirements accounted for a 20% variance in evoked postcesarean pain, which is similar to our findings [9]. They also demonstrated that the three questions have a sensitivity and specificity of 0.69 and 0.69, respectively, for identifying parturients in the top 20th percentile for activity-associated postcesarean pain. The ease of administering the pain rating questionnaire in the clinical setting makes it an attractive tool for identifying parturients at risk for severe postoperative pain.

In a follow-up study, the authors used the three pain rating questions to identify parturients predicted to have postoperative pain above the 80th percentile and randomly assigned them to a standard dose of spinal morphine (150 mcg) and an increased dose of spinal morphine (300 mcg). Parturients in the intervention group had decreased postoperative pain scores with movement at 24 h compared to the control group [18].

In our multivariate model, the pain reported on LAI did not predict mean postoperative pain with movement in the first 48 h after CD. Orbach-Zinger et al. reported a high sensitivity and specificity of LAI pain score as a predictor of severe acute postoperative pain. However, their study was limited to the first 24 h following surgery [13]. Their study also used an on-patient request analgesic protocol compared to a standardized scheduled analgesic regimen in our study. A more recent study by Nimmaanrat et al. showed a moderate correlation between LAI pain intensity and severity of pain with movement and at rest at 24 h after CD in patients who received a standardized analgesic regimen after surgery [19]. The pain experienced during LAI is biphasic. There is the initial pain on needle insertion, followed by pain on delivery of the local anesthetic into the tissues [20]. The second pain is usually more intense as pain receptors respond to chemical irritation and rapid tissue distension [21]. In our study, the biphasic character of pain on LAI was not a consideration. Other factors, such as the needle angle on injection, subdermal versus intradermal injection, and rate of injection, can all influence the pain experienced by the patient [22]. These factors can result in significant variability when rating pain on LAI and may reduce its reliability as a predictor of postoperative pain. The prediction of the severity of postoperative pain based on observation of a patient's response to a noxious stimulus (LAI), to our knowledge, has not been studied in patients having CD. In a study by Hunter et al. investigating the prediction of immediate postoperative pain by surgeons and anesthesiologists, they reported that physicians overestimated the degree of pain [23].

We acknowledge several limitations in our study. First, we did not assess sleep deprivation, anxiety, baseline mood, or other psychological states that could influence acute postoperative pain and opioid consumption. In addition, maternal satisfaction scores and pain levels upon discharge were not measured. Our findings may not be directly applicable to primary CD patients due to the significant proportion of participants with prior CDs in our study population. Although we aimed to include patients with chronic pain or substance abuse history, they constituted less than 10% of our study population, precluding a subgroup analysis. Furthermore, the anesthesiologists' predictions of pain severity were based on observed patient reactions to LAI, but we lacked standardized scoring criteria. We also did not adjust for the years of experience of the anesthesiologist. Finally, the overall pain scores in our study population were relatively low, likely due to the implementation of a multimodal analgesia regimen, which may not reflect the pain experiences of patients under different analgesic protocols.

## 5. Conclusions

In conclusion, this study demonstrates that a simple preoperative pain rating questionnaire combined with an anesthesiologist's prediction of postcesarean pain severity explains 27% of the variance in mean postoperative pain with movement. While this predictive model does not capture all factors influencing postcesarean pain, it provides a practical, clinically feasible tool for identifying patients at higher risk for significant postoperative pain. The findings support the potential utility of integrating patient-reported pain expectations and anesthesiologist assessments into individualized pain management strategies. Future research should investigate whether tailoring analgesic interventions based on these predictors improves pain control and patient outcomes following CD.

## Figures and Tables

**Table 1:** Demographic and obstetric data of the study cohort (*n* = 90).

Age (years)	31.48 ± 5.57
Body mass index (kg/m^2^)	35.78 ± 7.52
Race	
Caucasian	75 (83.3)
African American/black	10 (11.1)
Asian	5 (5.5)
Ethnicity	
Hispanic	8 (8.9)
Non-Hispanic	82 (91.1)
Gestational age (weeks)	38 [38–39]
Nulliparous	15 (16.6)
Indications for CD	
Breech	17 (18.9)
Previous CD	62 (68.9)
Other	11 (12.2)
QBL	612.5 [439–813]
Intraabdominal adhesions	8 (8.9)
History of chronic pain	5 (5.6)
History of substance abuse	4 (4.4)

*Note:* Values are expressed as mean ± SD, median (IQR), and number (%) as appropriate.

Abbreviations: CD = cesarean delivery; QBL = quantitative blood loss.

**Table 2:** Summary of predictor and outcome variables (*n* = 90).

	Mean
Expected postoperative pain	6.73 ± 1.69
Anticipated threshold for analgesia	6.08 ± 1.77
Perceived analgesic requirements	5.77 ± 2.22
Anesthesiologist prediction of pain severity	3.66 ± 2.21
Pain rating on local anesthesia infiltration	3.81 ± 2.63
Pain at rest at 6 h	2.74 ± 2.08
Pain with movement at 6 h	4.01 ± 2.54
Pain at rest at 24 h	2.73 ± 2.12
Pain with movement at 24 h	4.05 ± 2.22
Pain at rest at 48 h	2.50 ± 1.86
Pain with movement at 48 h	3.46 ± 2.05
Mean pain with movement	3.87 ± 1.95
Opioid intake (MME) at 24 h	26.38 ± 27.51
Opioid intake (MME) at 48 h	45.25 ± 39.92
First opioid request (hr)	8.68 ± 10.44

Abbreviation: MME = morphine milligram equivalent.

**Table 3:** Univariate model for mean pain with movement during first 48 h after CD.

Predictors	*β*	95% CI		
		Lower	Upper	*p* value
Expected postoperative pain (Q1)	0.40	0.155	0.624	0.0011
Anticipated analgesia threshold (Q2)	0.183	−0.052	0.418	0.1273
Perceived analgesic needs (Q3)	0.336	0.159	0.513	0.0002
Average of Q1, Q2, and Q3	0.642	0.352	0.932	< 0.0001
Average of Q1 and Q3	0.570	0.327	0.812	< 0.000
Anesthesiologist's prediction of pain severity	0.226	0.044	0.407	0.0147
Pain rating on LAI	0.221	−0.071	0.372	0.0040
Parity	−1.214	−2.295	−0.133	0.0277
History of cesarean section	0.387	−0.552	1.326	0.4189
Body mass index (kg/m^2^)	−0.028	−0.082	0.026	0.3097
Gestational age (weeks)	−0.139	−0.689	0.412	0.6215

Abbreviation: LAI = local anesthesia infiltration.

**Table 4:** Multivariate model for mean pain with movement during first 48 h after CD.

Predictors	*β*	95% CI		
		Lower	Upper	*p* value
Average of Q1, Q2, and Q3	1.204	0.636	1.772	< 0.0001
Anesthesiologist's prediction of pain severity	1.138	0.333	1.942	0.0056
Average of Q1, Q2, and Q3^∗^ prediction of pain severity	−0.157	−0.285	−0.029	0.0162
		*R* ^2^ = 0.27		

*Note:* Q1, expected postoperative pain; Q2, anticipated analgesia threshold; Q3, perceived analgesic needs.

^∗^Interaction term between the average of Q1, Q2, and Q3 and the anesthesiologist's prediction of postoperative pain severity.

## Data Availability

The data supporting this study's findings are available from the author with the permission of the University of Iowa Human Subjects Office (Institutional Review Board).

## Supporting Information

STROBE checklist Version 4.
